# Case report: Exploring under the tip of the iceberg: A case series of “self-limiting” multisystem inflammatory syndrome in children

**DOI:** 10.3389/fped.2022.1012582

**Published:** 2022-12-13

**Authors:** Alessandra Meneghel, Giorgia Martini, Marta Balzarin, Nadia Zabadneh, Michele Fastiggi, Francesca Tirelli, Francesco Zulian

**Affiliations:** ^1^ Pediatric Rheumatology Unit, Department for Women’s and Children’s Health, University Hospital of Padova, Padova, Italy; ^2^ Pediatric Unit, “San Bortolo” Hospital, Vicenza, Italy; ^3^ Pediatric Unit, “Ospedali Riuniti Padova Sud” Hospital, Schiavonia, Italy

**Keywords:** multisystem inflammatory syndrome in children (MIS-C), COVID-19, self-limiting disease, case report, SARS-COV-2

## Abstract

Multisystem inflammatory syndrome in children (MIS-C) is a serious condition triggered by SARS-COV-2 infection, characterized by persistent fever, multiorgan dysfunction, and increased inflammatory markers. It requires hospitalization and prompt treatment, with nearly 60% of the cases needing intensive care and 2% fatality rate. A wide spectrum of clinical characteristics and therapeutic approaches has been reported in MIS-C. We describe a series of four patients with MIS-C, defined according to the current case definitions, with a self-limiting course and no need for immunomodulatory treatment (“self-limiting MIS-C”). Few data about self-limiting MIS-C are available to date and no information on medium- and long-term outcome of this subset of patients has been reported. Although limited in size, our experience provides new insights into the MIS-C syndrome, highlighting an underestimated aspect of the disease that may have significant therapeutic implications.

## Introduction

Multisystem inflammatory syndrome in children (MIS-C) is a potentially life-threatening condition triggered by SARS-COV-2 infection (1). Since its first description (2, 3), huge effort has been made worldwide to better understand the pathogenesis and the clinical features of this novel entity to optimize therapeutic approaches in view of a favorable short- and long-term outcome. Different MIS-C case definitions have been provided so far (4–6), partly overlapping with each other, defining the picture of a severe condition, often requiring intensive care and close monitoring, mostly characterized by persistent fever with multiorgan involvement and increased inflammatory markers in the context of a proven previous exposure to SARS-COV-2 (RT-PCR, antigen test, or positive serology) with the exclusion of other possible infectious causes. Since COVID-19 can be asymptomatic or mild infection in children (7), the finding of a positive serological test for SARS-COV-2 is a confirmatory criterion in a suspected clinical setting for MIS-C.

Despite this general wide range of clinical manifestations, outcomes have been reported with nearly 60% of cases requiring intensive care (8, 9) and 2% fatality rate (8).

Because of the overlapping features with Kawasaki disease (KD) (1–3), MIS-C patients have been treated mainly with intravenous immunoglobulin (IvIg) and corticosteroids. This combination therapy has shown to be effective (10) although recent studies did not demonstrate superior evidence of combination therapy as compared to monotherapy (11). More recently, the American College of Rheumatology (ACR) provided revised recommendations about immunomodulatory treatment in MIS-C patients, in which a panel of experts agreed that “*in mild cases, after evaluation by specialists with expertise in MIS-C, some patients may be managed with only close monitoring without immunomodulatory treatment*” (12). Furthermore, some authors recently reported their experience with a multistep therapeutic strategy, according to different severities in clinical presentation of MIS-C patients, that demonstrated a favorable impact on MIS-C course, preventing intensive care unit (ICU) admission (9).

In the context of the wide variety of clinical features and course of MIS-C, we describe a series of four patients, diagnosed according to the most recent criteria (5, 6) with a self-limiting course characterized by spontaneous recovery and no need for immunomodulatory treatment (“self-limiting MIS-C”).

## Case series

Case 1: An 8-year-old healthy girl presented with prolonged fever (8 days) associated with abdominal pain and erythematous skin rash involving the face, the neck, and the upper trunk. She had a history of COVID-19 family exposure 6 weeks earlier. She was not vaccinated for SARS-COV-2. Physical examination revealed the girl to be suffering with elective abdominal pain on the right lower abdomen, mild tachycardia, and hypotension [heart rate (HR) 126 bpm and blood pressure (BP) 98/59 mmHg]. Blood test showed the following: platelets (PLT) 129,000/mm^3^; C-reactive protein (CRP) 127 mg/L; procalcitonin (PCT) 6.7 μg/L; D-dimer 847 μg/L; fibrinogen 4.82 g/L; and normal electrolytes, hepatic and renal function tests, pancreatic enzymes, troponin I (TpnI), and B-type natriuretic peptide (BNP). Viral serologies were negative for Epstein–Barr virus (EBV), adenovirus, cytomegalovirus (CMV), herpes virus, and parvovirus-b19 (PVB19), and positive for SARS-COV-2 IgG s-RBD (92.72 kAU/L). Blood and stool cultures were negative; fecal calprotectin was normal. Electrocardiogram (EKG) and echocardiography were normal. Abdominal ultrasound (US) showed marked and extended thickening of the last ileal loop's wall, with heterogenicity of the adjacent adipose tissue and multiple lymphadenomegalies. Intravenous ceftriaxone was administered empirically for 5 days. She showed complete resolution of abdominal pain and skin rash on day 2 after admission and fever on day 5. Repeat abdominal US confirmed decrease of the ileal wall thickening and resolution of the other findings. She was discharged on day 8. At 1-month follow-up visit, she was asymptomatic and abdominal US showed complete resolution of terminal ileitis. At 6-month follow-up visit, she was persistently asymptomatic.

Case 2: A 5-year-old healthy boy not yet vaccinated for SARS-COV-2 presented with a 6-day history of fever associated with abdominal pain, diarrhea, and bilateral conjunctivitis. He had COVID-19 infection 4 weeks earlier. On physical examination, bulbar bilateral conjunctivitis, mild systolic heart murmur, mild tachycardia, and hypotension were present (BP 108/64 mmHg and HR 110 bpm). Blood tests showed the following: PLT 179,000/mm^3^; CRP 64.2 mg/L; PCT 1.73 μg/L; fibrinogen 5.24 mg/L; D-dimer 239 μg/L; BNP 129 ng/L [normal value (n.v.) < 100]; TpnI 38 ng/L (n.v. < 16); and normal electrolytes, hepatic, and renal function tests. Serology and PCR for adenovirus, parvovirus, CMV, and EBV was negative. Viral serological test found positivity for SARS-COV-2 IgG s-RBD (86.2 kAU/L). EKG showed nonspecific changes in ventricular repolarization. Echocardiography and chest x-ray were normal. Blood and stool cultures were negative. Oral amoxicillin/clavulanic acid was started. One day after admission, fever disappeared, and the gastrointestinal symptoms improved. Blood tests confirmed reduction in inflammatory markers and normalization of BNP and Tpnl. He was discharged on day 3. After 1 week, he was asymptomatic with normal EKG and echocardiography. At 1-month and 6-month follow-up visits, he was asymptomatic and blood tests were normal.

Case 3: 6 weeks after COVID-19 infection, a 12-year-old previously healthy boy, not vaccinated for SARS-COV-2 virus, presented with fever for 6 days associated with diffuse skin rash, diffuse abdominal pain, and diarrhea. Physical examination showed elective pain on the right lower abdomen and polymorphous erythematous skin rash on trunk, upper and lower limbs; and mild increased heart rate with normal blood pressure (BP 115/75 mmHg and HR 122 bpm). Blood tests showed the following: white blood cells (WBC) 14,010/mm^3^ [neutrophils (N) 12,130/mm^2^, lymphocyte (L) 1,110/mm^3^], D-dimer 337 μg/L, fibrinogen 9.72 g/L, normal BNP and TpnI, CRP 122.6 mg/L, erythrocyte sedimentation rate (ESR) 42 mm/h, and PCT 1.74 μg/L. Chest *x*-ray, EKG, and echocardiography were normal. Abdominal US showed marked thickening of the last ileal loop with multiple reactive lymphadenomegalies. No therapy was started. One day after admission, fever disappeared with complete resolution of mucocutaneous and gastrointestinal involvement on day 2. Repeated blood test showed: CRP 23.84 mg/L, ESR 25 mm/h; BNP 84 mg/L. He was discharged on day 3. At 1 month and 6-months follow-up visit he was asymptomatic; blood tests, EKG and echocardiography were normal.

Case 4: An 8-year-old healthy girl presented with 3-day lasting fever associated with persistent pain on the right side of the abdomen. She was not vaccinated for SARS-COV-2 and she got COVID-19 infection 4 weeks earlier. Blood tests showed the following: WBC 10,500/mm^3^ (N 8,400/mm^3^), PLT 214,000/mm^3^, CRP 65 mg/L, ESR 73 mm/h, D-dimer 472 μg/L, fibrinogen 5.09 g/L, TpnI normal, N-terminal brain natriuretic peptide (NT-BNP) 745 ng/L, alanine aminotransferase (ALT) 56 U/L, and aspartate aminotransferase (AST) 49 U/L. SARS-COV-2 IgG s-RBD resulted positive (63.97 kAU/L). Stool and blood cultures were negative. Abdominal US showed moderate thickening of the last ileal loop's wall, with hyperechogenicity of the adipose tissue and multiple reactive lymphadenomegalies. EKG and echocardiography were normal. No therapy was started. On day 1 after admission, fever disappeared with improvement of abdominal pain, which completely resolved on day 3. She was discharged on day 7. At 1-month and 6-month follow-up visits, she was asymptomatic and blood tests were normal.

## Discussion

In 2 years (April 2020–2022), we observed 54 cases of classical MIS-C and 4 patients (7.5%) with clinical features of MIS-C but with a “self-limiting” course (Table 1). These patients had a mean age of 8.25 years (range 5–12) and presented with fever (mean 5.75 days duration before diagnosis) associated with mucocutaneous involvement (75%), gastrointestinal complaints (100%), and cardiovascular involvement (50%). None of the patients needed hemodynamic support or pediatric intensive care admission. All documented a previous COVID-19 exposure, with positive SARS-COV-2 antibody testing (IgG s-RBD) in three cases. In one case, serological test for SARS-COV-2 was not done because of a previous documented infection 4 weeks earlier. All patients displayed a rapid resolution: three of them had spontaneous regression of fever within 24 h since admission, and cutaneous, cardiovascular, and gastrointestinal complaints subsided in some days for all. Along with the clinical findings, inflammatory and cardiac markers showed spontaneous decrease too. The absence of severe cardiovascular involvement, the favorable clinical course, and the overall good condition of the children allowed a conservative management with just clinical observation, brief hospitalization, and no need for immunomodulatory treatment.

**Table 1:** Characteristics and outcome of patients with self-limiting MIS-C.

	Clinical presentation	Laboratory results	Imaging findings	COVID-19 history	SARS-COV-2 IgG (n.v. < 1)	PICU	Therapy	Hospitalization (days)	Total days of fever	Outcome 6 month
Patient 1 8 years old Female No comorbidities	8 days fever; abdominal pain; erythematous skin rash	PLT 129,000/mm^3^, CRP 127 mg/L, PCT 6.7 μg/L, D-dimer 847 μg/L, fibrinogen 4.82 g/L	Normal EKG and echocardiogram. terminal ileitis on abdominal US	Intrafamily exposure 6 weeks earlier	92.72 kAU/L	None	Ceftriaxone IV	8	13	CR
Patient 2 5 years old Male No comorbidities	6 days fever; abdominal pain; non-bloody diarrhea; bilateral conjunctivits	PLT 179,000/mm^3^, CRP 64.2 mg/L, PCT 1.73 μg/L, fibrinogen 5.24 mg/L, D-dimer 239 μg/L, BNP 129 ng/L, troponin I 38 ng/L	EKG: nonspecific changes in ventricular repolarization; normal echocardiogram	COVID-19 infection 4 weeks earlier	86.2 kAU/L	None	Amoxicillin/acid clavulanic p.o.	3	7	CR
Patient 3 12 years old Male No comorbidities	6 days fever; erythematous skin rash; bilateral conjunctivitis; abdominal pain; diarrhea	WBC 14,010/mm^3^, N 12,130/mm^3^, L 1,110/mm^3^, CRP 122.6 mg/L, PCT 1.74 μg/L; fibrinogen 9.72 g/L, d-dimer 337 μg/L	Normal EKG and echocardiogram. terminal ileitis on abdominal US	COVID-19 infection 4 weeks earlier	Not done	None	None	7	7	CR
Patient 4 8 years old Female No comorbidities	3 days fever; abdominal pain	WBC 10,500/mm^3^, N 8,400/mm^3^, PLT 214,000/mm^3^, CRP 65 mg/L, fibrinogen 5.09 g/L, D-dimer 472 μg/L, ALT 56 U/L, AST 49 U/L, NT-BNP 745 ng/L	EKG and echocardiogram normal; terminal ileitis on abdominal US	COVID-19 infection 4 weeks earlier	63.97 kAU/L	None	None	7	4	CR

PICU, pediatric intensive care unit; PLT, platelets; CRP, C-reactive protein; PCT, procalcitonin; BNP, brain natriuretic peptide; ALT, alanine aminotransferase; AST, aspartate aminotransferase; WBC, white blood cells; N, neutrophils; L, lymphocytes; EKG, electrocardiogram; US, ultrasound; CR, complete remission; IV, intravenous; p.o., per os; n.v., normal value.

There are few data about similar experience in the literature. Ouldali et al. in their comparative study on immunomodulatory treatment for MIS-C reported, in the supplemental contents, five patients with spontaneous resolution of fever with no need for immunomodulators (10). All patients presented with fever, with an average duration of 4.4 days, and mucocutaneous and cardiovascular involvement. Three patients presented with gastrointestinal and respiratory complaints and two with central nervous system (CNS) involvement. Indeed, two patients needed pediatric intensive care unit (PICU) referral. Considering laboratory parameters, the average values of inflammatory markers (CRP, PCT, ferritin), D-dimer, and cardiac enzymes (TpnI, BNP, NT-BNP) were higher in this cohort than in our series. Although the authors reported resolution of fever after 9.2 days on average, they did not provide any other information about the following course and outcome.

In other studies, the declared percentages of MIS-C patients spontaneously recovering with only supportive care ranged between 6% and 22% (11–13). Davies et al. reported that 12/78 (15%) patients who recovered spontaneously from MIS-C without immunomodulatory therapy. 42% of them received invasive ventilation and 58% required inotropes. The authors did not find any statistically significant difference in the short-term outcome between those treated and untreated. No data about the long-term outcome in this subgroup of patients were reported.

Finally, McArdle et al., in the BATS study, stated that 39/614 MIS-C cases (6.3%) did not receive any immunomodulatory treatment and only 15% needed organ support in the intensive care setting. They found that the average level of inflammatory markers decreased more rapidly in the group of treated patients than in those not treated. However, no information about the long-term outcome was given.

Although our experience is limited in size, we showed that after 6 months, patients with self-limiting MIS-C did not experience any significant sequelae despite no specific treatment. The observation that some patients can recover spontaneously from MIS-C is probably not surprising if we consider that self-limited cohorts of KD have been also reported (14). On the other hand, the fact that MIS-C may have a self-limiting course raises important considerations about diagnosis and management of this subgroup of patients. Firstly, it is possible that self-limiting MIS-C is underestimated because milder forms may not require hospitalization and, due to the overlapping features with other febrile conditions of infancy, may not be properly recognized. Therefore, we need more information about the long-term outcome of this subset of patients to verify whether a missed treatment in a self-limiting disease may increase the risk of sequelae, as shown in self-limiting KD (15).

The ACR recently suggested that mild cases of MIS-C, after evaluation by experts in MIS-C, can require only close clinical monitoring without immunomodulatory treatment (12). In our series, the absence of laboratory and instrumental findings of cardiac involvement was the key point for a conservative approach, although in other cohorts in which a self-limited course has been reported, cardiovascular dysfunction was described (10–13).

Although we have seen a decline in MIS-C cases in the latest COVID-19 waves, we do not have available data to define if this reflects the impact of vaccinations or of the variants of the virus itself.

Finally, considering the variable course of the disease and the spread of SARS-COV-2 vaccination in the pediatric population, we do need more selective criteria to guide treatment decision in MIS-C patients. This may allow avoiding aggressive immunomodulatory treatments when unnecessary.

## Data Availability

The raw data supporting the conclusions of this article will be made available by the authors, without undue reservation.

## References

[1] Feldstein LR Rose EB Horwitz SM Collins JP Newhams MM Son MBF Multisystem inflammatory syndrome in U.S. children and adolescents. N Engl J Med. (2020) 383(4):334–46. 10.1001/jama.2021.2091 32598831 PMC7346765

[2] Riphagen S Gomez X Gonzalez-Martinez C Wilkinson N Theocharis P. Hyperinflammatory shock in children during COVID-19 pandemic. Lancet. (2020) 395:1607–8. 10.1016/S0140-6736(20)31094-1 32386565 PMC7204765

[3] Verdoni L Mazza A Gervasoni A Martelli L Ruggeri M Ciuffrdda M An outbreak of severe Kawasaki-like disease at the Italian epicentre of the SARS-CoV-2 epidemic: an observational cohort study. Lancet. (2020) 395:1771–8. 10.1016/S0140-6736(20)31103-X 32410760 PMC7220177

[4] Royal College of Health Paediatrics and Child (RCPCH). Guidance: paediatric multisystem inflammatory syndrome temporally associated with COVID-19. Available at: https://www.rcpch.ac.uk/sites/default/files/2020-05/COVID-19-Paediatric-multisystem-%20inflammatory%20syndrome-20200501.pdf (Accessed March 10, 2022).

[5] World Health Organization (WHO). Multisystem inflammatory syndrome in children and adolescents with COVID-19. WHO reference number: WHO/2019-nCoV/Sci_Brief/Multisystem_Syndrome_Children/2020.1. Available at: https://www.who.int/publications/i/item/multisystem-inflammatory-syndrome-in-children-and-adolescents-with-covid-19 (Accessed March 10, 2022).

[6] CDC Multisystem inflammatory syndrome in children (MIS-C) associated with coronavirus disease 2019 (COVID-19). Available at: https://emergency.cdc.gov/han/2020/han00432.asp (Accessed March 10, 2022).

[7] Di Chiara C Cantaratutti A Costenaro P Donà D Bonfante F Cosma C Long-term immune response to SARS-COV-2 infection among children and adults after mild infection. JAMA Netw Open. (2022) 5(7):e2221616. 10.1001/jamanetworkopen.2022.21616 35816313 PMC9280400

[8] Abrams JY Oster ME Godfred-Cato S Bryant B Datta SD Campbell AP Factors liked to severe outcomes in multisystem inflammatory syndrome in children (MIS-C) in the USA: a retrospective surveillance study. Lancet Child Adolesc Health. (2021) 5(5):323–31. 10.1016/S2352-4642(21)00050-X 33711293 PMC7943393

[9] Brisca G Consolaro A Caorsi R Pirlo D Tuo G Campanello C Timely recognition and early multi-step autoinflammatory therapy may prevent ICU admission of patients with MIS-C: proposal for a severity score. Front Pediatr. (2021) 9:783745. 10.3389/fped.2021.783745 34988039 PMC8721096

[10] Ouldali N Toubiana J Antona D Javouhey E Madhi F Lorrot M Association of intravenous immunoglobulins plus methylprednisolone vs immunoglobulins alone with course of fever in multisystem inflammatory syndrome in children. JAMA. (2021) 325:855–64. 10.1001/jama.2021.0694 33523115 PMC7851757

[11] McArdle AJ Vito O Patel H Seaby EG Shah P Wilson C Treatment of multisystem inflammatory syndrome in children. N Engl J Med. (2021) 385:11–22. 10.1056/NEJMoa2102968 34133854 PMC8220965

[12] Henderson LA Canna SW Friedman KG Gorelik M Lapidus SK Bassiri H American college of rheumatology clinical guidance for multisystem inflammatory syndrome in children associated with SARS-CoV-2 and hyperinflammation in pediatric COVID-19: version 3. Arthritis Rheumatol. (2022) 74(4):e1–20. 10.1002/art.42062 35118829 PMC9011620

[13] Davies P Lillie J Prayle A Evans C Griffiths B du Prè P Association between treatments and short-term biochemical improvements and clinical outcomes in post-severe acute respiratory syndrome coronavirus-2 inflammatory syndrome. Ped Crit Care Med. (2021) 22(5):e285–93. 10.1097/PCC.000000000002728 PMC8096187 33767074

[14] Whittaker E Bamford A Kenny J Kaforou M Jones CE Shah P Clinical characteristics of 58 children with a pediatric inflammatory multisystem syndrome temporally associated with SARS-COV-2. JAMA. (2021) 324(3):259–69. 10.1001/jama.2020.10369 PMC7281356 32511692

[15] Takahashi T Sakakibara H Morikawa Y Miura M. Development of coronary artery lesions in indolent Kawasaki disease following initial spontaneous defervescence: a retrospective cohort study. Ped Rheumatol. (2015) 13(44):1–8. 10.1016/S2352-4642(18)30293-1 PMC4632407 26530040

